# Evidence for an ancient whole genome duplication in the cycad lineage

**DOI:** 10.1371/journal.pone.0184454

**Published:** 2017-09-08

**Authors:** Danielle Roodt, Rolf Lohaus, Lieven Sterck, Riaan L. Swanepoel, Yves Van de Peer, Eshchar Mizrachi

**Affiliations:** 1 Department of Genetics, Forestry and Agricultural Biotechnology Institute, University of Pretoria, Private bag X20, Pretoria, South Africa; 2 Centre for Bioinformatics and Computational Biology, Genomics Research Institute, University of Pretoria, Private bag X20, Pretoria, South Africa; 3 Department of Plant Biotechnology and Bioinformatics, Ghent University, Gent, Belgium; 4 VIB Center for Plant Systems Biology, Gent, Belgium; 5 Bioinformatics Institute Ghent, Ghent University, Ghent, Belgium; National Cheng Kung University, TAIWAN

## Abstract

Contrary to the many whole genome duplication events recorded for angiosperms (flowering plants), whole genome duplications in gymnosperms (non-flowering seed plants) seem to be much rarer. Although ancient whole genome duplications have been reported for most gymnosperm lineages as well, some are still contested and need to be confirmed. For instance, data for ginkgo, but particularly cycads have remained inconclusive so far, likely due to the quality of the data available and flaws in the analysis. We extracted and sequenced RNA from both the cycad *Encephalartos natalensis* and *Ginkgo biloba*. This was followed by transcriptome assembly, after which these data were used to build paralog age distributions. Based on these distributions, we identified remnants of an ancient whole genome duplication in both cycads and ginkgo. The most parsimonious explanation would be that this whole genome duplication event was shared between both species and had occurred prior to their divergence, about 300 million years ago.

## Introduction

Whole genome duplications (WGDs) have been prevalent during the evolutionary history of flowering plants, and have even been linked to their origin as well as their fast rise to ecological dominance [1–3]. Furthermore, although the duplication of entire genomes is mostly regarded as an evolutionary dead-end [4–7], it has been proposed that, in times of rapid environmental change, WGDs can confer an important evolutionary advantage [8–11]. This is, for instance, suggested by the fact that many angiosperm lineages show evidence for independent WGD events around the Cretaceous-Paleogene (K-Pg) extinction ~66 million years ago (Mya) [11, 12].

Contrary to the many WGD events recorded for angiosperms, the history of the non-flowering gymnosperms paints a very different picture. Although far fewer gymnosperm species exist today compared to the angiosperms, and as such many lineages containing evidence for WGD events could have been lost, polyploidy events, ancient or more recent, in these seed plants seem rare. Thus far, *Welwitschia mirabilis* is the only gymnosperm showing evidence for a relatively recent WGD event [13, 14], possibly also overlapping the K-Pg boundary. In any case, this event occurred more recently than the divergence of *Welwitschia* from its closest relative, *Gnetum* (135–110 Mya) [15–17], the genome of which shows no sign of a WGD [13]. Furthermore, *Ephedra*, the third Gnetales genus, also lacks evidence of WGD events [13], excluding very recent duplication events that resulted in the widespread polyploidy seen in extant species of this genus [18–20]. Li *et al*. [13] also provided evidence for independent ancient WGDs in the conifer lineage that may have coincided with the more ancient Permian-Triassic boundary, ~250 Mya. Similarly, as with the angiosperms, these conifer-specific WGDs might have contributed to the survival and success of the conifer lineage during periods of drastic environmental change [13]. The same study found evidence for an ancient WGD in the *Ginkgo* lineage, attributing it to the ancient WGD event proposedly shared by all seed plants [1]. Clear remnants of WGDs in cycads were not uncovered, likely due to the dearth of available public EST data [21], resulting in insufficient resolution to call an ancient WGD event in this lineage.

The cycads were widespread during the Jurassic–Cretaceous, reaching their greatest diversity ~200–65 Mya [22–24]. Today, however, only a mere 348 extant species in ten genera remain [25]. The dramatic decrease in diversity was likely due to challenges such as at least three mass extinction events, as well as the arrival of, and major competition from, the angiosperms. Although the lineage itself dates back ~270 million years, most extant cycad species originated much more recently, most likely within the past 65 million years [22, 26, 27]. Therefore, the popular referral to cycads as living fossils is not entirely accurate, as the lineage itself is ancient but most species originated relatively recently. Their continued survival is somewhat paradoxical, as they have particularly slow growth and cannot compete with the fast growing, rather short-lived angiosperms. Here, we confirm that cycads have undergone an ancient WGD and show that this event was likely shared with *Ginkgo biloba*, preceding the divergence of these lineages.

## Results and discussion

**Fig 1:**
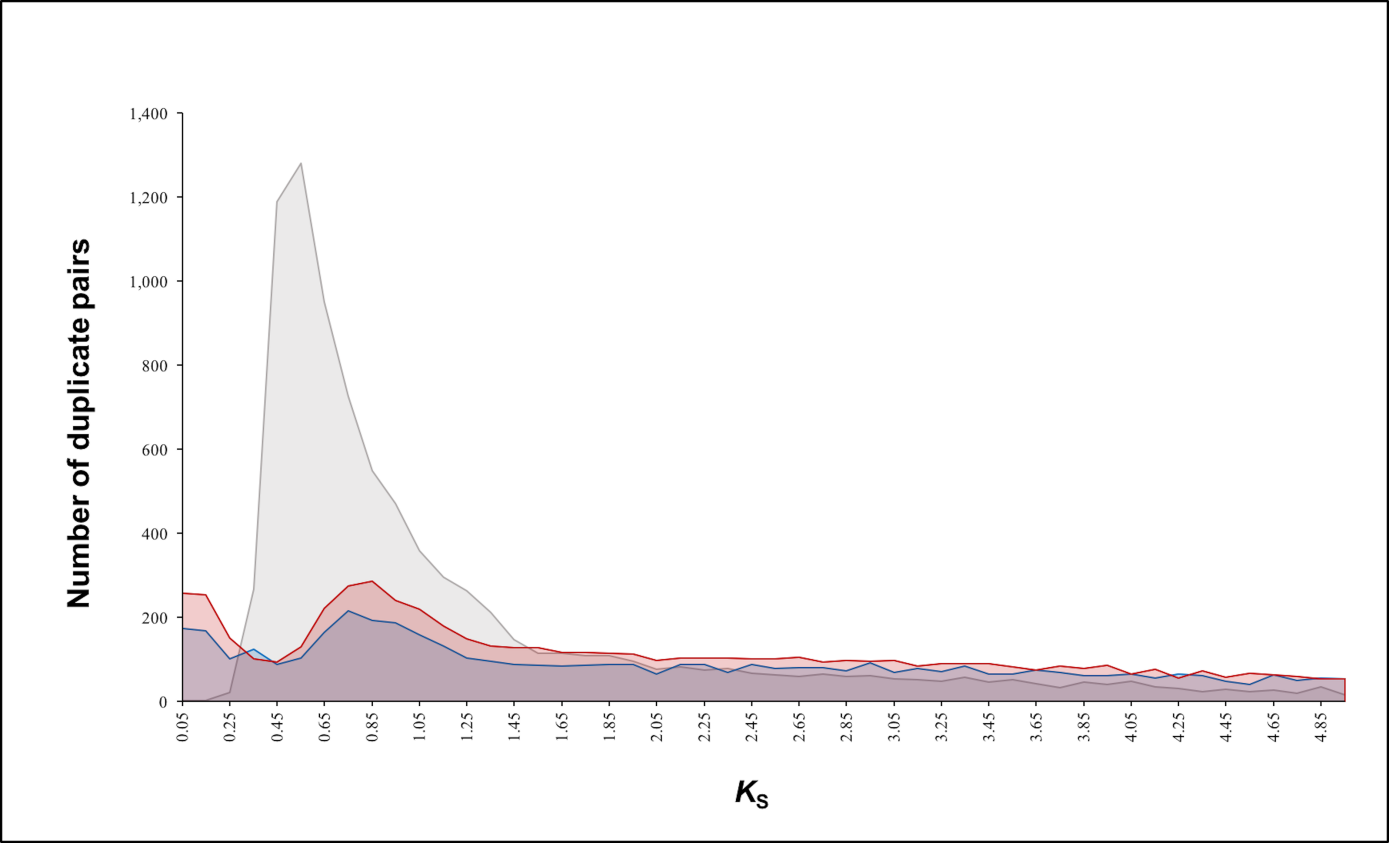
*K*_S_ age distributions for *Ginkgo biloba* and *Encephalartos natalensis* transcriptomes. The red graph represents the age distribution of duplicates (in the transcriptome) of *G*. *biloba*, while the blue graph represents the age distribution of duplicates of *E*. *natalensis*. The graph in grey denotes the *K*_S_ distribution for one-to-one orthologs of *G*. *biloba* and *E*. *natalensis*.

We sequenced transcriptome data from two tissues (see Materials and Methods) of representatives of both *Encephalartos natalensis* (a native cycad species from the Kwazulu-Natal province of South Africa) and *Ginkgo biloba*, and assembled high quality low-redundancy transcriptomes [28]. The *E*. *natalensis* and *G*. *biloba* assemblies contained 22,204 and 23,845 transcripts with average sequence lengths of 1,097 and 1,259 bases and average GC contents of 44.23 and 42.52%, respectively. Based on age distributions of paralogs inferred from synonymous substitutions per synonymous site, or so-called *K*_S_ distributions [29], a distinct peak with a median *K*_S_ of ~0.8 was identified for *E*. *natalensis* (Fig 1), a clear signature of an ancient WGD event. *G*. *biloba* showed a similar *K*_S_ distribution, and also contained a peak at a *K*_S_ of ~0.8 (Fig 1). This distribution is consistent with data reporting the presence of a WGD event in the evolutionary history of ginkgo [13]. Since the *K*_S_ peaks for both *E*. *natalensis* and *G*. *biloba* were at similar *K*_S_ values (Fig 1), this could suggest either a similar timeframe for both WGDs or a shared WGD event in the ancestor of the two lineages.

**Fig 2:**
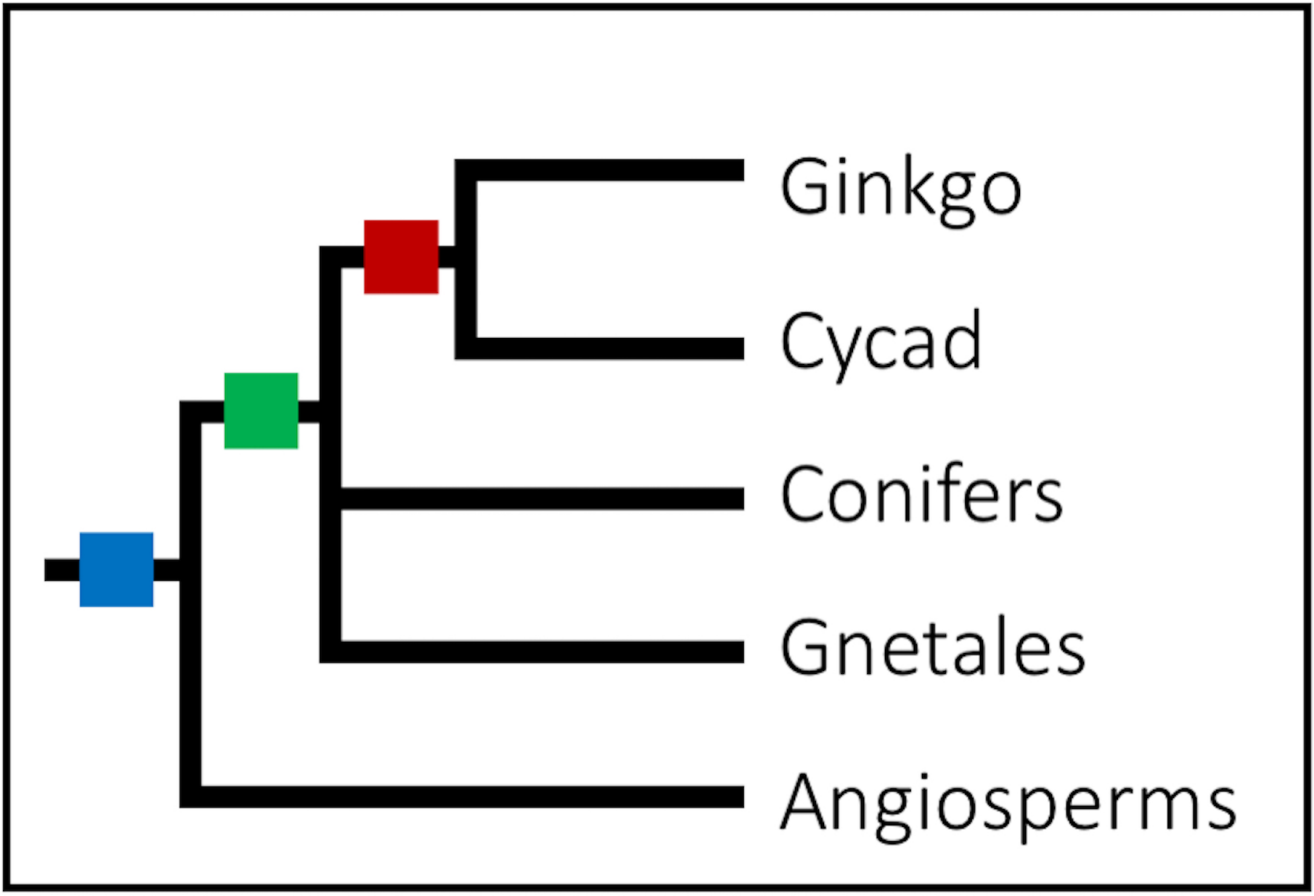
Possible locations for the whole genome duplication uncovered for *Encephalartos natalensis* and *Ginkgo biloba*. Cycad and ginkgo share a common ancestor and are sister to the ancestor of Gnetales and conifers [17, 30, 34, 35, 37, 39]: the inferred polyploidy event could have occurred in the common ancestor of the cycad and gingko lineages (red square), the gymnosperm ancestor (green square), or in the seed plant ancestor (blue square). Note that the position of Gnetales remains uncertain (see text for details).

We built a *K*_S_ age distribution of one-to-one orthologs between *E*. *natalensis* and *G*. *biloba* to investigate whether the duplication peaks identified in both indicated a shared WGD. Assuming similar substitution rates in the two lineages [30], the time of speciation seems to be slightly younger than the polyploidy events in both *G*. *biloba* and *E*. *natalensis* (Fig 1). Cycad and ginkgo are thought to have diverged from one another, and from the other gymnosperms, ~330–270 Mya [16, 17, 27, 31, 32]. Although several studies suggest that cycads diverged earliest during gymnosperm evolution, with the ginkgo lineage following not long thereafter [16, 26, 31–33], others show cycads and ginkgo as having diverged from a common ancestor [17, 34–37] (Fig 2). In either case, the more parsimonious explanation would be to assume that the duplication peaks observed in both lineages represent a shared event, rather than independent ancient duplications that have occurred early in the evolution of both lineages. However, the correct phylogenetic placement of this WGD event is uncertain. The WGD might represent the ancient seed-plant-specific WGD assumed to have occurred ~340 Mya [1], but could also be gymnosperm-specific or nested within the gymnosperms (Fig 2) [38].

Due to the slow substitution rates of most of the gymnosperm taxa [13, 31, 39], these ancient WGD events in the lineages of cycad and ginkgo (Fig 1), and conifers [13] are still discernible as distinct peaks in their *K*_S_ distributions (Fig 1, Suppl. Fig S3 in [13]). Slow substitution rates result in gradual genomic change, conserving the remnants of ancient events in the genomes of these species. Because these lineages separated from one another hundreds of millions of years ago, absolute dating of these WGD events is challenging, but they likely indicate a shared event in a common ancestor (Fig 2).

It should be noted that recently a draft genome for *G*. *biloba* was presented, in which the authors claimed to have found evidence for two WGD events based on the detection of two different peaks in a *K*_S_ distribution of paralogs [40]. However, we think their *K*_S_ distributions should be interpreted with caution, since the youngest peak, corresponding to a WGD estimated between 147 and 74 Mya [40], is likely artificial and the result of thresholds used to consider genes as duplicates rather than as identical. Furthermore, the inferred WGD dates as obtained by Guan *et al*. [40] should be considered unrealistic. For instance, the authors assume the older genome duplication to be between 735–515 Mya, which would predate the origin of land plants. As we have shown in the current study, the WGD in ginkgo is probably only slightly older (0.2 *K*_S_) than the divergence between cycads and ginkgo (Fig 1).

If the WGD detected here did not occur in the ancestor of the cycads and ginkgo, but earlier in the gymnosperm evolution (green or blue squares in Fig 2), we would expect to see remnants of this event in the Gnetales. Yet the complete absence of an ancient peak in the Gnetales remains difficult to explain. One reason why such evidence is lacking might be the faster rate of evolution in Gnetales, compared to other gymnosperms [39, 41, 42], resulting in any traces of ancient polyploidies to be lost. On the other hand, the placement of the Gnetales within the gymnosperms remains elusive. While different molecular markers have placed them within the conifer lineage (the ‘Gnepine’ and ‘Gnecup’ hypotheses), sister to the conifers (the ‘Gnetifer’ hypothesis), or sister to all other gymnosperms (the ‘Gnetales-sister’ hypothesis) [30, 34, 36, 43–45], certain morphological traits even place them closer to the angiosperms, or as a basal seed plant [46, 47]. Therefore, if the WGD event detected here indeed represents an ancient gymnosperm or seed-plant WGD (see [38]), the lack of evidence for WGDs in the Gnetales, except the more recent one in *Welwitschia*, and the difficulty in resolving their exact phylogenetic placement, could suggest an evolutionary history that is different from the other gymnosperms. In conclusion, despite the fact that early seed plant evolution remains problematic with respect to both phylogenetic relationships and relative and absolute dating of WGD events, we here provide conclusive evidence that cycads have also undergone an ancient WGD.

## Materials and methods

Materials from *Encephalartos natalensis* and *Ginkgo biloba* were obtained from plants grown at the Manie van der Schijff Botanical Garden at the University of Pretoria, South Africa. Leaflets and rachis samples were collected separately from *E*. *natalensis*, while mature leaf and stem tissue were sampled from three male *G*. *biloba* trees. RNA was extracted using a standard CTAB RNA extraction method [48], followed by a clean-up step using the Qiagen RNeasy Plus Mini Kit. RNA was sent for sequencing at Novogene, China, generating 150-bp libraries.

RNA-seq libraries from these tissues were used to construct *de novo* transcriptome assemblies for *E*. *natalensis* (66,583,315 paired end reads; 20 Gbp) and *G*. *biloba* (98,762,732 paired end reads; 29.6 Gbp). The *de novo* assemblies were constructed with Velvet v1.2.10 [49] followed by Oases v0.2.08 [50] with a k-mer size of 101, retaining only contigs larger than 200 bases. The redundancy of the assemblies was removed with the Evidential gene pipeline and only the primary transcript of each loci was used in further analyses [51].

TransDecoder v.3.0.0 was used to predict coding regions in the transcriptomes of *E*. *natalensis* and *G*. *biloba* [52], after which the 19,991 and 23,845 longest coding and peptide sequence transcripts were selected for the species, respectively. *K*_S_ age distributions for *E*. *natalensis* and *G*. *biloba* were constructed as described previously [29]. Briefly, to construct the paranome an all-against-all BLASTP search was performed of all the longest transcripts with an E-value cutoff of 1 × 10^−10^, followed by gene family construction and prediction using the mclblastline pipeline (v10-201, http://micans.org/mcl) [53]. Each gene family was aligned using MUSCLE (v3.8.31). To obtain *K*_S_ estimates for all pairwise comparisons in gene families, maximum likelihood estimation was performed using the CODEML program of the PAML package (v4.4c) [54, 55]. Gene families were then subdivided into subfamilies for which *K*_S_ estimates between members did not exceed a value of 5. To correct for the redundancy of *K*_S_ values (a gene family of *n* members produces *n*(*n*–1)/2 pairwise *K*_S_ estimates for *n*–1 retained duplication events), a phylogenetic tree was constructed for each subfamily using PhyML [56] under default settings. For each duplication node in the resulting phylogenetic tree, all *m K*_S_ estimates between the two child clades were added to the *K*_S_ distribution with a weight of 1/*m* (where *m* is the number of *K*_S_ estimates for a duplication event), so that the weights of all *K*_S_ estimates for a single duplication event sum up to one. One-to-one orthologous pairs between *E*. *natalensis* and *G*. *biloba* were created by performing a reciprocal best hit BLASTP analysis of the longest translated transcripts from one species against the other. Valid orthologous pairs were then identified as having at least 30% identity over 150 amino acids.

## Accession numbers

Raw reads of both transcriptomes are available at the National Center for Biotechnology Information (https://www.ncbi.nlm.nih.gov/) under the submission number SUB2337915.
